# Employment Intention and Associated Factors of Nursing Graduates: A Structural Equation Model

**DOI:** 10.1155/jonm/7402874

**Published:** 2025-03-30

**Authors:** Xinmin Zhang, Yongai Zhang, Jiming Han, Yanhui Jiao, Jinpei Chen, Miao Chu, Zitong Guo

**Affiliations:** ^1^The Medical School, Yan'an University, Yan'an, Shaanxi, China; ^2^The Faculty of Nursing and Rehabilitation, Xi'an Medical University, Xi'an, Shaanxi, China; ^3^The Operating Theater, The First Affiliated Hospital of Xi'an Jiaotong University, Xi'an, Shaanxi, China

**Keywords:** employment intention, latent class analysis, nursing graduates, psychological capital, Roy's adaptation model

## Abstract

**Objective:** To assess the associated factors of employment intention among contemporary nursing graduates.

**Background:** In the postepidemic era, the shortage of nurses and the decline in the employment rate have garnered significant attention globally. It is crucial to evaluate the factors associated with the employment intentions of nursing graduates to address the issue of nurse shortage. So far, existing research on the employment intentions of nursing graduates in China has mainly focused on examining the relationship between individual factors and employment intentions. However, there is a notable lack of systematic research grounded in scientific nursing theories that predicts employment intentions and related factors by constructing structural equation models to validate the interrelationships among variables.

**Methods:** A total of 1332 nursing graduates were selected from 15 undergraduate colleges and universities in Shaanxi Province, Northwest China, using cross-sectional multistage cluster sampling. A model was constructed, and variables including human capital, learning satisfaction, social support, professional values, psychological capital, and employment intention were measured through structured questionnaires. Descriptive analysis was conducted using SPSS 26.0, while AMOS 24.0 was utilized for the verification and analysis of structural equation modeling.

**Results:** The final model explained 20.2% of the total variation of nursing graduates' employment intention. Learning satisfaction (*B* = 0.417, *p* < 0.001), social support (*B* = −0.101, *p* < 0.05), professional values (*B* = 0.630, *p* < 0.001), and psychological capital (*B* = 0.594, *p* < 0.001) significantly influence employment intention. The total effects of learning satisfaction and psychological capital on employment intention were (0.225 and 0.283), direct effects of (0.129 and 0.235), and indirect effects of (0.096 and 0.048), respectively.

**Conclusion:** The employment intentions of nursing graduates are influenced by various factors, among which psychological capital is the most significant.

**Implications for Nursing Management:** The education departments related to nursing talent cultivation should continuously explore the effectiveness of vocational education, adopt various social support methods, optimize the undergraduate nursing teaching model, and improve the learning satisfaction and human capital level of nursing graduates. This will enhance their psychological capital and help them establish a correct professional value system, thereby increasing the employment intentions of nursing graduates.

**Summary**

• **Reporting Method:** Strengthening the Reporting of Observational Studies in Epidemiology (STROBE) criteria were used to report the survey results.

## 1. Background

Nursing work is an important cornerstone of the healthcare industry. As the largest professional group in the healthcare system, nurses play a crucial role in maintaining and promoting the health of people, as well as in preventing sudden public health emergencies and coping with related national and global health events. However, the shortage of nurses remains a major issue globally [1, 2], with high turnover and mobility rates being common phenomena in the nursing workforce [3, 4]. In 2018, it was reported that there was a global shortage of approximately 5.9 million nurses, with about 5.3 million of the shortage concentrated in low- and middle-income countries. The aging workforce is also threatening the stability of nursing populations in some regions [5]. China also faces a severe shortage of nurses, with only about 4 registered nurses per 1000 people by the end of 2023 [6], which is significantly lower than in Europe (7.93), the Americas (8.34) [5], Japan (12.85) [7], and Australia (14.07) [8].

China has released the National Nursing Development Plan (2021–2025), which points out that the main contradiction in the nursing field is between the demand for nursing services from the public and the relatively insufficient supply [9]. The aging and shortage of the nursing workforce are major issues faced by the health workforce sector [10, 11]. One key solution is to retain nursing graduates. Nursing graduates are a major part of the future nursing workforce, ensuring that choosing a nursing career upon employment can guarantee the effective development of the health sector. Employment is the most fundamental aspect of people's livelihoods, and the employment of college graduates is a significant matter that relates to development stability and the well-being of the populace. In China, employment work still faces many prominent contradictions and issues, especially the severe challenges brought about by the COVID-19 pandemic on the employment of nursing professionals. The employment rate of many college graduates has significantly declined, leading to phenomena such as waiting for jobs, unemployment, and a lack of motivation to seek employment. However, the employment situation of college graduates in China serves as an important basis for the allocation of educational resources, assessment of educational quality, and planning of enrollment [12]. A study in China shows that the occurrence of COVID-19 has led to a low intention among nursing students to pursue a long-term career in nursing. Among 1020 nursing students, 45.4% indicated that they might leave the nursing profession in the future [13]. Similarly, the situation of nursing students entering the nursing profession in Iran is also not optimistic. Haririan et al. [14] conducted a survey of 284 nursing students regarding their intention to leave the nursing profession, and nearly one-third of the students expressed a strong desire to leave.

However, up to now, mainstream research on the employment intentions of nursing graduates in China has mainly examined the relationship between individual factors and employment intention, lacking systematic research to explore the interrelationships among various factors and integrate them into a model of employment intention. Therefore, it is necessary to conduct a systematic investigation and research on the employment intentions of Chinese nursing graduates based on scientific theories.

### 1.1. Literature Review

The employment of nursing graduates is affected by a combination of internal and external elements, involving factors such as individuals, schools, and families. The learning satisfaction of college students is one of the important aspects of higher education evaluation and management. It is the most direct assessment of their perceptions after participating in higher education [15]. Social support refers to the emotional experience of individuals regarding the support they receive from others or the respect they feel from others [16]. Previous studies have confirmed the correlation between nursing graduates' employment intentions and their learning satisfaction [15], as well as social support [17]. Research indicates that the professional values of nursing graduates were an important factor influencing their employment intentions [18]. Nursing professional values are the cornerstone for establishing nursing beliefs, and the cultivation of professional values among nursing students has become an important aspect of nursing education [19]. Psychological capital refers to a positive psychological condition exhibited by individuals during the process of growth and development [20]. Psychological capital has been shown to be correlated with the employment intentions of nursing graduates [21–23]. Human capital refers to the total sum of knowledge, skills, and health that individuals possess, which are formed through formal education and social practice, and have socioeconomic value [24]. Previous research has shown a positive correlation between the human capital and the employment outcomes of college students [25].

### 1.2. Structural Equation Model

This study is based on Roy's Adaptation Model, which emphasizes the individual as a holistic adaptive system, where behavior is a response to internal and external environmental stimuli, manifested in two forms: adaptive responses and ineffective responses. Adaptive responses contribute to the individual's survival, growth, reproduction, and self-realization, while ineffective responses hinder individual development [26]. In the employment context of nursing graduates, various stimuli from the internal and external environment can influence their behavioral choices. Positive stimuli may promote adaptive behaviors, leading graduates to choose and persist in a nursing career. Conversely, negative stimuli may result in ineffective behaviors, prompting them to leave the nursing profession. Therefore, the employment choices and behavioral outcomes of nursing graduates are influenced by stimuli in the internal and external environment.

This theory provides a framework for understanding the employment behavior of nursing graduates. Overall, Roy categorizes the stimuli in the internal and external environments that can affect individuals into three types: focal stimuli, contextual stimuli, and residual stimuli. Focal stimuli are the stimuli that individuals currently face and must respond to, often related to survival conditions, immense pressure, etc.; contextual stimuli are all other stimuli that can have a positive or negative impact on the behavior caused by primary stimuli, such as social interactions, work, and learning environments; residual stimuli refer to factors that may influence current behavior, but whose effects are uncertain, often related to a person's character, attitudes, beliefs, culture, and values [27]. Nursing graduates face a focal stimulus during employment, which is role transition, specifically the shift from nursing students to nurses. According to the theory of role function mode extended from Roy's Adaptation Model, the transition of roles means that individuals need to free themselves from the influence of the behavioral patterns and psychological characteristics of the previous role and develop a complete set of behavioral patterns and psychological characteristics required for the new role. They must adjust their state to enter the new role in order to better accomplish the tasks assigned by the new role. This role transition process involves multiple dimensions, including role cognition, behavioral performance, emotional attitudes, abilities and skills, as well as social support [27, 28]. These dimensions are closely related to several exogenous variables and observed variables in this study, which are integrated as follows.

The role cognition dimension integrates observed variables such as self-awareness, social awareness, nursing professionalism, and the role of nursing work within professional values. This dimension focuses on graduates' understanding of the nurse role and their identification with their professional identity. The behavioral performance dimension integrates observed variables such as adaptability, self-efficacy, and resilience. This dimension reflects the behavioral adaptability and coping abilities exhibited by graduates during the role transition process. The emotional attitude dimension integrates observed variables such as hope, optimism, student expectations, student complaints, and student loyalty. This dimension focuses on the emotional experiences and attitude changes of graduates during the role transition process. The abilities and skills dimension integrates observed variables such as occupational skills, interpersonal skills, and personal traits. This dimension emphasizes the specific skills and personal characteristics required by graduates during the role transition. The social support dimension integrates the exogenous variable of social support, focusing on the external support that graduates receive during the role transition process.

This study integrates role transition with exogenous variables such as human capital, learning satisfaction, social support, professional values, and psychological capital into a theoretical framework. Social support and learning satisfaction are classified as contextual stimuli in the employment process of nursing graduates, treated as exogenous variables. Professional values and psychological capital are seen as manifestations of nursing professional ethical value recognition and positive psychological state, respectively, and are classified as residual stimuli in the employment process of nursing graduates, treated as mediating variables. Additionally, nursing students with higher human capital tend to have a higher level of professional identity and greater employment intention [29]. Therefore, treating human capital as exogenous variables, previous research indicates that behavioral intention is a direct predictor of behavior [30]. Therefore, this study treated employment intention, the best predictive factor for employment behavior, as the outcome variable and include it in the theoretical framework along with the aforementioned variables.

**Figure 1:**
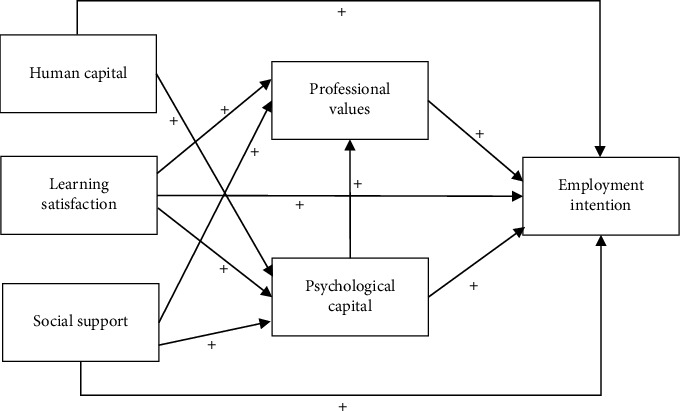
Theoretical framework.

In addition, based on existing research evidence, the pathways between various variables are proposed. Studies indicate that the higher the level of professional values among nursing students, the more willing they are to engage in the nursing profession [31, 32]. The higher the psychological capital, the greater the willingness of nursing students to pursue a nursing career [33, 34]. Furthermore, there is a positive correlation between professional values and psychological capital [35], which serve as mediating variables. Learning satisfaction, as an exogenous variable, is positively correlated with professional values [36], psychological capital [37], and employment intention [15]. Similarly, social support, as an exogenous variable, has also been shown to have a positive correlation with professional values [38, 39], psychological capital [38, 39], and employment intention [17]. Research shows that there is a positive correlation between human capital and psychological capital [40]. The pathways generated by the aforementioned variables are included in this model, and based on Roy's Adaptation Model theory and previous research, the following hypothetical model is proposed (Figure 1).

### 1.3. Research Purpose

This study aims to construct a structural equation model of the employment intentions of Chinese nursing graduates and validate it, elucidating the relationships between variables. The goal is to provide a theoretical basis and reference for the Ministry of Education, universities, and employers to carry out nursing education and employment-related guidance and support work.

## 2. Methods

### 2.1. Overview

This study adopts a cross-sectional survey design to investigate the nursing graduates from 15 regular undergraduate colleges in Shaanxi Province, China. It uses structural equation modeling to analyze the relationships between various variables and employment intentions.

### 2.2. Research Subjects

This study selected research subjects using a multistage cluster sampling method. The time frame was from April 2024 to June 2024. Initially, the study focused on the northwest region within the seven major geographical regions of China: East China, South China, Central China, North China, Northeast China, Northwest China, and Southwest China. Among the five provinces in the northwest region, Shaanxi Province was selected as the sampling unit. There are a total of 15 undergraduate colleges and universities in Shaanxi Province that offer four-year nursing programs, including 7 public institutions (Xi'an Medical University, Yan'an University, Shaanxi University of Chinese Medicine, Ankang University, Shangluo University, Xi'an Jiaotong University, and Air Force Medical University) and 8 private institutions (Xi'an International Studies University, Xi'an Peihua University, Xi'an Siyuan University, Xijing University, Xi'an Fanyi University, Xi'an Innovation College of Yan'an University, Xi'an Jiaotong University City College, and Shaanxi University of International Trade and Commerce). This study used the WenJuanXing platform to distribute the survey uniformly, including nursing graduates from these 15 schools meet the inclusion criteria as research subjects. The specific inclusion and exclusion criteria are as follows.

Inclusion criteria is defined as follows: nursing graduates who have completed 24 weeks or more of clinical practice in hospitals and those who are aware of the survey content and are willing to cooperate in completing the questionnaire. Since the data collection for this study utilized social media and digital methods, we established the exclusion criteria as inability to access the Internet, or not using relevant social media.

In addition, according to the sample size calculation method of structural equation model, the baseline sample size should be 15–20 times the number of predictor variables [41]. In this study, there were 22 measured variables. Calculated at 20 times the number of measured variables, the calculation formula is (4 + 5+3 + 4+4 + 2) ∗ 20 = 440. Therefore, this study aims to collect at least 440 samples to meet the requirements. It is estimated that there will be around 2000 nursing graduate graduates in Shaanxi Province in 2024, with 1902 graduates responding to the questionnaire. During the data collection process, some nursing graduates were unwilling to discuss their true thoughts on employment, leading to careless and perfunctory responses. Therefore, based on the criteria for excluding invalid questionnaires: participants who answered the survey but whose response time was less than $\bar{x}-1.96s$ ($\bar{x}$ = 486.34, *s* = 72.64), those with identical values for multiple options, and those with contradictory answers to reverse questions were excluded. Ultimately, 1332 valid questionnaires were used for this analysis. Among them, there are 575 students from public universities and 757 students from private universities, both of which meet the minimum sample size requirements.

### 2.3. Measuring Tools

#### 2.3.1. College Student Human Capital Scale

This study used the College Student Human Capital Scale as the evaluation tool [42], comprising four dimensions: adaptability, occupational skills, interpersonal skills, and personal traits, with a total of 16 items. The Likert 5-level scoring method was used. Cronbach's *α* coefficient of the scale was 0.930 [40], and in this study, Cronbach's *α* was 0.905.

#### 2.3.2. Learning Satisfaction Questionnaire

This study used the Learning Satisfaction Questionnaire developed by Yan [43] as the evaluation tool. The questionnaire consists of 5 dimensions, totaling 41 items, including school image, student expectations, perceived quality, student complaints, and student loyalty. The scale uses a Likert 5-level scoring method. Cronbach's *α* coefficient of this questionnaire was 0.969 [43], and in this study, Cronbach's *α* was 0.976.

#### 2.3.3. Perceived Social Support Scale

The Perceived Social Support Scale was developed and compiled by Zimet et al. [44], and translated into Chinese by Wang [45]. It consists of 12 items across 3 dimensions: family support, friend support, and other support. The scale uses a Likert 5-level scoring method. Cronbach's *α* coefficient for the Chinese version of the scale was 0.88 [45], while in this study, Cronbach's *α* was 0.967.

#### 2.3.4. Nursing Professional Values Scale

The Nursing Professional Values Scale compiled by Yeun, Kwon, and Ahn [46] was used to measure the professional values of nursing graduates. This scale comprises four dimensions: self-awareness, social awareness, nursing professionalism, and the role of nursing work, totaling 25 items. The Likert 5-level scoring method was used. Cronbach's *α* coefficient for the Chinese version of the scale was 0.87 [47], and in this study, Cronbach's *α* was 0.933.

#### 2.3.5. Positive Psychological Capital Questionnaire

The Positive Psychological Capital Questionnaire was developed by Zhang, Zhang, and Dong [48], which includes four dimensions: self-efficacy, resilience, hope, and optimism, totaling 26 items. It uses Likert 7-level scoring method, with higher scores indicating better psychological capital among nursing graduates. Cronbach's *α* coefficient of this questionnaire was 0.90 [48], and in this study, Cronbach's *α* was 0.933.

#### 2.3.6. Nursing Employment Intention Scale

The nursing employment intention scale developed by Cowin [49] was translated into Chinese by the authors of this study to measure the intention to pursue a nursing career. The scale includes two dimensions: staying in the nursing profession and leaving the nursing profession, with a total of six items. The Likert 8-level scoring method was used. Cronbach's *α* coefficient of the original scale was 0.90 [49], while in this study, Cronbach's *α* coefficient was 0.885.

### 2.4. Data Collection

The researchers of this project contacted the persons in charge of nursing departments at various universities to gain their agreement. After obtaining their consent, we introduced the purpose, significance, data collection methods, and other information about the research to them. We also obtained their agreement to train them as investigators for this research project. Our team applied a unified set of guidelines for research investigators to conduct training for the implementation of the project. The investigators from the collaborating universities organized online meetings for nursing graduates at their respective schools, and they introduced the purpose, significance of the questionnaire, and instructions for filling it out. After ensuring that the research subjects were informed of the above information, electronic questionnaires were distributed to students via the WeChat platform. The questionnaire's first page included an informed consent form, and if the research subjects selected “no,” the survey would automatically end. Each respondent was restricted to answering the questionnaire only once from a single IP address, and all questions had to be answered before submitting the questionnaire to ensure no missing data.

### 2.5. Data Analysis

In this study, data collection was completed using the WenJuanXing platform and exported directly. The collected data were then analyzed using SPSS 26.0 software for descriptive statistics and correlation analysis. The reliability of the scale was evaluated using Cronbach's *α* coefficient. Quantitative data were represented using ($\bar{x}\pm s$), while qualitative data were described using frequency and composition ratios.

This study uses AMOS 24.0 to validate and modify the model. The structural equation modeling is a comprehensive statistical method that includes two main parts: the measurement model and the structural model. First, in the measurement model, the validity and reliability of the observed variables and latent variables are tested through confirmatory factor analysis (CFA), including construct reliability (CR > 0.7), average variance extracted (AVE > 0.5), and discriminant validity. The Pearson correlation coefficient is used to compare with discriminant validity to examine the quality of discriminant validity. Second, in the structural model, path analysis is used to determine the relationships between the latent variables. The model fit is tested using the comparative fit index (CFI > 0.90), normed fit index (NFI > 0.90), Tucker–Lewis index (TLI > 0.90), goodness-of-fit index (GFI > 0.90), and root mean square error of approximation (RMSEA < 0.07). Bootstrap ML analyzes the indirect effects, direct effects, total effects, and the significance of statistical results among various variables. Under normal distribution and large sample sizes, the output results of model estimation using maximum likelihood (ML) estimation are more stable [50]. The bias-corrected confidence intervals at 95% were selected, with a resampling count of 5000 times. Two-sided *p* values less than 0.05 were considered significant.

In this study, a large sample size exceeding the minimum sample size was use, which can reduce the probability of Type II errors. Sufficient data can make model estimates more stable and reduce the impact of sample fluctuations on model parameter estimates. Additionally, a larger sample size can meet assumptions such as asymptotic normality.

Furthermore, this study reduces the potential Type I errors that may arise from a large sample size by ensuring the quality of the research design, adjusting analysis strategies, and optimizing model specifications, among other methods. Specifically, this includes using reliable and valid measurement tools to collect data, ensuring the accuracy of variable measurements, and reducing Type I errors caused by measurement errors; employing the bootstrap method to obtain multiple subsamples through resampling, estimating model parameters, and utilizing its stability to reduce Type I errors; and simplifying the model based on theoretical frameworks and research objectives to avoid excessive complexity that could lead to overfitting.

### 2.6. Ethical Considerations

This research has received approval from the Ethics Committee at Xi'an Medical University (approval number: XYYJSLS2023001). The participants voluntarily enrolled in this investigation and provided signed informed consent before the study.

## 3. Results

### 3.1. Characteristics

A total of 1332 nursing graduates participated in this study, with an average age of 21.89 ± 2.70. Among them, there were 173 males (13%) and 1159 females (87%). 623 students (46.8%) voluntarily chose nursing, 467 students (35.1%) were influenced by others to choose nursing, and 242 students (18.2%) transferred to the nursing major. 251 students (18.8%) planned to pursue a long-term nursing career after graduation, 137 students (10.3%) planned to pursue a master's degree in nursing, 247 students (18.5%) definitely stated they will change careers in a few years, 588 students (44.1%) were undecided and may change careers, 88 students (6.6%) were determined to change careers immediately after graduation, and 21 students (1.6%) planned to take a postgraduate entrance exam in a different field. In the choice of employment regions, 715 students chose metropolis (53.7%), 439 students chose small and medium-sized cities (33.0%), 65 students chose county towns (4.9%), 6 students chose townships (0.5%), and 107 students (8.0%) had a neutral attitude toward this choice.  Table 1 illustrates all the general characteristics of the research subjects.

### 3.2. Measurement Model

The measurement model includes six latent variables and 22 measured variables. In the validation process, the standard factor load values of each measurement variable were all higher than 0.45 [51], as shown in Table 2. Furthermore, CFA was conducted on the measurement model. The results indicate that the AVE values corresponding to the factors were all greater than 0.5, and the composite reliability (C.R) values were all higher than 0.7 [50], demonstrating good convergent validity of the data (Table 2).

According to Pearson correlation coefficient, it can be seen that there was a positive correlation between employment intentions and human capital, learning satisfaction, social support, professional values, and psychological capital (*r* = 0.297, *p* < 0.001; *r* = 0.358, *p* < 0.001; *r* = 0.300, *p* < 0.001; *r* = 0.342, *p* < 0.001; *r* = 0.378, *p* < 0.001) (Table 3). Finally, a discriminant validity test was conducted on the measurement model (Table 3). The results show that the Pearson correlation coefficients between variables were all less than the square root of AVE, indicating that the model has good discriminant validity [52].

### 3.3. Structural Equation Model

**Figure 2:**
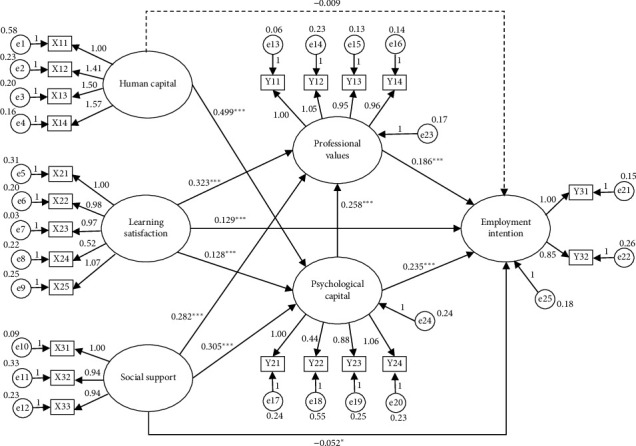
Structural equation model of employment intentions of nursing graduates. Solid arrows represent statistically significant paths, while dashed arrows indicate nonsignificant paths. ⁣^∗^*p* < 0.05, ⁣^∗∗∗^*p* < 0.001. X11: adaptability, X12: occupational skills, X13: interpersonal skills, X14: personal traits, X21: school image, X22: student expectations, X23: perceived quality, X24: student complaints, X25: student loyalty, X31: other support, X32: family support, X33: friend support, Y11: self-awareness, Y12: social awareness, Y13: nursing professionalism, Y14: the role of nursing work, Y21: self-efficacy, Y22: resilience, Y23: hope, Y24: optimism, Y31: staying in the nursing profession, Y32: leaving the nursing profession.

This study's theoretical framework (Figure 1) comprises 11 paths. Based on the theoretical framework, a hypothetical model was constructed, and the fit indices of the hypothetical model are fully presented in Table 4. According to the modification indices from the software, the model was further refined, and the final model is shown in Figure 2, which includes a total of 11 paths. The fit of the modified model is better than that of the hypothetical model, and thus, the modified model is considered acceptable, as detailed in Table 4.

The parameter estimates of the modified structural equation model for the employment intentions of nursing graduates are shown in Table 5. Among them, the explanatory power of employment intentions for nursing graduates through learning satisfaction, social support, professional values, and psychological capital was 20.2%. The explanatory power of psychological capital through human capital, learning satisfaction, and social support was 64.6%. The explanatory power of professional values through learning satisfaction, social support, and psychological capital was 56.6%.

### 3.4. Effect

In this study, psychological capital has the greatest impact on the outcome variable of employment intention, human capital has the greatest impact on the mediating variable of psychological capital, and social support has the greatest impact on the mediating variable of professional values. In the hypothesized paths of this study, the path from human capital to employment intention is not statistically significant, while the direct and overall effects of the other 10 paths are statistically significant. The indirect effects of human capital, learning satisfaction, social support, and psychological capital on employment intention are also statistically significant, as detailed in Table 6.

## 4. Discussion

### 4.1. Analysis of the Current Employment Intentions of Nursing Graduates

The shortage of talent in the nursing industry is a highly concerning issue for global health services. Enhancing the social recognition of the nursing profession is an important aspect of higher nursing education in China, and the employment of nursing graduates is a key factor in evaluating the effectiveness of nursing talent training. It analyzes the general characteristics of nursing graduates included in this study, nearly one-third of nursing graduates are willing to engage in the nursing profession long term, a result slightly lower than the 34.6% found in Lai's study [53]. The distribution of employment expectations among nursing graduates is uneven, which is similar to the findings of Wang's research [54]. The average score for employment intention is lower than the 5.25 ± 1.69 points reported in Ahn's study [55]. Similarly, in Choi's research, the average score for nursing students' long-term career intentions was 5.56 ± 0.71 points [56], which is significantly higher than the results of this study. Analyzing the reasons, it may be because the salaries, career development opportunities, work intensity and environment, nurse–patient relationships, and social status of nurses in China and South Korea are all different. The nursing profession in China still needs to be further valued and developed. Based on the above, it is recommended that government departments actively build a high-quality employment guidance service system, improve policies on salary, title evaluation, training, and further education, promote the implementation of the “academic certificate + vocational skills certificate” system for nursing professionals, and strengthen public education to guide society in establishing a correct view of employment. This includes creating a favorable public opinion atmosphere and an inclusive social environment that emphasizes “no profession is superior or inferior, and labor deserves respect” and “employment at the grassroot level is equally commendable,” in order to enhance the employment intentions of nursing graduates and avoid the waste of educational resources and the loss of nursing talent.

### 4.2. Fit of the Employment Intention Structural Model for Nursing Graduates

This study was based on Roy's Adaptation Model. After validating and modifying the structural model, the fit degree of the structural equation model for employment intentions among nursing graduates was at a good level. Such independent variables as learning satisfaction, social support, professional values, and psychological capital can directly influence employment intention, with these variables explaining 20.2% of the variance in employment intention, which is lower than the 37.8% explanatory power of various factors on nurses' retention willingness found in Lee's study [57]. The analysis suggests that the reason may be that the population in this study consists of Chinese nursing graduates, while Lee's study focused on a group of Korean nurses. The differences in region and population have led to the varying results between the two studies. Additionally, there were indirect effects of human capital, learning satisfaction, social support, and psychological capital on employment intention. The variables that directly influence psychological capital were human capital, learning satisfaction, and social support, which together explain 64.6% of the variance in psychological capital. Furthermore, learning satisfaction, social support, and psychological capital serve as direct influencing factors on professional values, accounting for 56.6% of the explanation rate for professional values.

### 4.3. The Relationships Between Variables in the Structural Equation Model of Employment Intention of Nursing Graduates

The results of this study show that the learning satisfaction and social support of nursing graduates can not only directly influence their employment intentions but also have an indirect impact through psychological capital and professional values. The research proves that a high level of learning satisfaction is related to students' positive behavioral intentions, meaning that satisfaction has a positive impact on student retention rates [15]. The correlation between learning satisfaction and psychological capital has been confirmed in a previous study, which indicates that learning satisfaction is positively correlated with psychological capital; learning satisfaction can help students reduce academic stress and enhance their psychological capital [37]. Furthermore, studies show that learning satisfaction is positively correlated with professional values, high learning satisfaction reflects students' sense of experience and fulfillment during the educational process, and students with high learning satisfaction tend to have a greater love for their school and major [36]. Social support has been shown to have a positive impact on employment intentions [17]. Additionally, research indicates that social support is positively correlated with psychological capital [58]; nursing students with higher levels of social support will directly influence their mental health and resilience, indirectly affecting their academic outcomes and employment [59, 60]. Social support from family and friends has been identified as one of the most important factors in maintaining students' motivation for success and focusing on academic requirements [38]. Consistent with Zhao's research, studies show that social support is positively correlated with professional values [61]. The variable of human capital also indirectly influences employment intention through psychological capital. Research shows that college students with a high level of human capital have greater employment advantages, a more positive employment mindset, and are better able to cope with the transition during this period, leading to higher psychological capital. This indicates a positive correlation between human capital and psychological capital [62]. In addition, when human capital is relatively weak, developing psychological capital can enhance employment intention [62]. At the same time, professional values and psychological capital have a positive impact on employment intention, which is consistent with previous research findings [31–34]. Therefore, when providing employment guidance education to nursing graduates, universities should fully recognize the key role of psychological capital and professional values education. University teachers can enhance students' psychological capital levels by regularly organizing group activities, such as team competitions and extracurricular physical training. Education on the values of the nursing profession is an important aspect of higher nursing education. University teachers can actively guide students during their time at school, such as by organizing screenings of nursing-related documentaries and sharing sessions with outstanding nurses, to help students establish a good professional value system, ultimately aiming to increase the employment intentions of nursing graduates.

In the results of this study, the path between social support and employment intention shows a negative impact, which contradicts previous research findings [17, 63]. This may be due to the widespread phenomenon of only children in Chinese families [64] and the increase in economic income, leading parents and family members to support and tolerate their children's decision not to seek employment [65]. Furthermore, previous studies indicate that from the perspective and culture of East Asian societies, most college students tend to choose their majors based on filial piety and respect, often referring to the suggestions given by parents or elders [66]. In reality, many nursing students do not like their major or the nursing profession they may pursue in the future [67]. Similarly, among the nursing students surveyed in this study, more than half chose the nursing major due to external influences or were redirected to the nursing program, indicating that nursing is not their ideal major, and thus, they are unwilling to work in the nursing field upon graduation. However, after the indirect effects of psychological capital and professional values, social support ultimately has a positive impact on employment intention. This also highlights the crucial role of professional values and psychological capital in enhancing the employment intentions of nursing graduates. Research has found that enhancing the level of social support among nursing graduates helps to improve their perseverance and self-efficacy, thereby increasing their psychological capital [38, 68]. Therefore, although the relationship between social support and employment intention in this study is negative, improving social support contributes to raising the psychological capital level of nursing students, and psychological capital is the most significant factor influencing employment intention in this study. Additionally, among the direct effects on employment intention, social support is the smallest. Therefore, expanding social support remains an intervention measure that nursing managers can consider to enhance the employment intention of nursing graduates. Higher education institutions should implement effective interventions to improve the employment quality of nursing graduates under the perspective of social support, such as adjusting talent training programs and actively providing emotional and informational support. Families need to change their traditional perceptions of the profession and provide respectful support, while the government should adjust employment strategies and offer relevant employment-related subsidies as much as possible.

In this study, the direct path from human capital to employment intention is not statistically significant, which differs from previous research [11, 69]. This may be because the study focuses on four-year undergraduate nursing students and does not include three-year nursing students. The research found that nursing students with higher education levels have career expectations that do not match the realities of the nursing profession. Students need to invest heavily in their human capital during their undergraduate studies in order to transition to other fields, and there is no correlation between high levels of human capital and their intention to seek employment [67]. In addition, this study found that among the influences on psychological capital, the effect of human capital is the largest, and psychological capital has the greatest impact on employment intention. The indirect effect of human capital on employment intention through psychological capital is statistically significant, consistent with Zhao's research [62]. This indicates that in student education, managers still need to focus on enhancing the human capital of nursing students. The enhancement of human capital requires attention to the improvement of college students' overall quality and the strengthening of their professional knowledge and skills. As an important institution for cultivating students' human capital, universities should focus on the development of students' human capital to increase the return on human capital. Teachers should innovate teaching models and improve educational programs, such as emphasizing the cultivation of nursing students' professional knowledge and practical abilities and regularly organizing clinical internships and classroom quizzes.

Finally, in this study, the path from psychological capital to employment intention was found to be statistically significant, consistent with previous research [33, 34], which indicates that positive psychological capital has a significant impact on the career preparation behaviors of nursing students. Nursing students with higher psychological capital were generally more willing to pursue a nursing career. Notably, professional values play a mediating role in the path from psychological capital to employment intention. Nursing graduates with higher psychological capital also tend to have a higher level of professional values, which aligns with Zheng's research findings [70]. Additionally, this study indicates that learning satisfaction directly and indirectly affects employment intention. This is consistent with the previous research findings [15, 36, 37]. Nursing graduates with higher learning satisfaction have a stronger employment intention. The research shows that the elements of teacher instruction and student interpersonal relationships are important factors contributing to learning satisfaction [71]. It is recommended that university teachers collect feedback from students about the classroom during the teaching process and establish a good relationship with students that is both teacher-student and friendly. Universities should focus on cultivating students' interpersonal skills, provide venues for social interaction, and encourage students to actively participate in beneficial and healthy academic exchanges or clubs, in order to enhance their learning satisfaction and ultimately increase the employment intentions of nursing graduates.

### 4.4. Limitations

This study has certain limitations. First, the representativeness and richness of the sample need to be improved. The sample in this study comes from nursing graduates of 15 general higher education institutions in Shaanxi Province, Northwest China, while there are about 300 universities in China offering a four-year nursing program [72]. The comprehensive personal situations of nursing graduates vary, so it was recommended that future studies expand the types and range of samples to enhance the generalizability and persuasiveness of the research results. Second, due to the limitations of cross-sectional survey research in terms of temporal relationships and causal relationships, the derivation of relationships between variables in this study relies on theory and previous research. It was suggested that future studies conduct longitudinal or interventional research on the factors influencing the employment intentions of nursing graduates to further explore and predict the factors affecting employment intentions.

## 5. Conclusion

This study was based on Roy's Adaptation Model and previous research findings to construct a structural equation model of the employment intentions of nursing graduates. The model explains 20.2% of the total variance in the employment intentions of nursing graduates and analyzes the effect sizes of various variables on employment intention, as well as the mediating roles of professional values and psychological capital in this process.

## 6. Implications for Nursing Management

The employment issues faced by nursing graduates are not only a phase problem for the students themselves but also a significant concern related to the construction of the nursing workforce and the advancement of the nursing industry. The stable employment of nursing students is essential for the rapid development of the nursing workforce and the continuous optimization of healthcare services. This study provides new ideas for nursing managers to take relevant measures to improve the employment intention of nursing graduates. Considering the impact of professional value, psychological capital, learning satisfaction, human capital, and social support on employment intention, nursing managers should pay attention to these factors and implement effective strategies to enhance the employment intentions of nursing graduates. For example, first, universities should strengthen nursing career development education and establish a phased, comprehensive, and distinctive career planning and employment guidance system for nursing. They should provide targeted employment guidance services. Graduates should be guided to reasonably adjust their employment expectations, accurately identify their career positioning, and actively seek employment with a healthy, positive, and rational mindset, thereby enhancing students' social support, professional values, and psychological capital levels. At the same time, it is important to optimize infrastructure services, such as improving public teaching facilities like libraries, specialized teaching facilities like laboratories, network resources like campus internet, and living facilities like cafeterias and sports fields, to comprehensively ensure the quality of students' campus life and improve their learning satisfaction. Additionally, it is recommended that universities organize career planning competitions for graduates, helping them improve their career planning and employment capabilities through competition, thus improving their human capital levels. Improving the employment intentions of nursing graduates can ensure that healthcare talent does not flow away, contributing to the long-term development of public health. Nursing managers should pay full attention to this issue.

## Figures and Tables

**Table 1:** General characteristics (*n* = 1332).

Factors	Groups	*n*	Frequency (%)	Mean (SD)
Gender	Male	173	13	
	Female	1159	87	

Age	≤ 18	9	0.7	21.89 ± 2.70
	19–21	392	29.4	
	≥ 22	931	69.9	

University types	Public institutions	575	43.2	
	Private institutions	757	56.8	

Origin of student	City	336	25.2	
	Rural area	996	74.8	

Why choose the nursing major?	Voluntary choice	623	46.8	
	Influenced by others	467	35.1	
	Transferred to the nursing major	242	18.2	

Internship satisfaction	Very dissatisfied	12	0.9	
	Not satisfied	27	2.0	
	General	385	28.9	
	Satisfied	567	42.6	
	Very satisfied	341	25.6	

The pressure of the nursing qualification exam	Heavy pressure	191	14.3	
	Moderate pressure	814	61.1	
	Mild pressure	267	20.0	
	No pressure	60	4.5	

Work plan after graduation	Long-term engagement in the nursing profession	251	18.8	
	Pursue a master's degree in nursing	137	10.3	
	Definitely change careers in a few years	247	18.5	
	Undecided and may change careers	588	44.1	
	Determined to change careers	88	6.6	
	Pursue a master's degree in other majors	21	1.6	

Employment area selection	Metropolis	715	53.7	
	Small and medium-sized cities	439	33.0	
	County town	65	4.9	
	Township	6	0.5	
	It does not matter	107	8.0	

**Table 2:** Verification results of confirmatory factor analysis.

Latent variable	Item	Factor load (FL)	Average variance extracted (AVE)	Construct reliability (CR)
Human capital	X11	0.483	0.654	0.879
	X12	0.776		
	X13	0.813		
	X14	0.852		

Learning satisfaction	X21	0.762	0.760	0.939
	X22	0.821		
	X23	0.959		
	X24	0.589		
	X25	0.810		

Social support	X31	0.962	0.791	0.919
	X32	0.870		
	X33	0.905		

Professional values	Y11	0.935	0.840	0.954
	Y12	0.806		
	Y13	0.850		
	Y14	0.847		

Psychological capital	Y21	0.863	0.658	0.880
	Y22	0.469		
	Y23	0.828		
	Y24	0.879		

Employment intention	Y31	0.924	0.938	0.885
	Y32	0.756		

*Note:* X11: adaptability; X12: occupational skills; X13: interpersonal skills; X14: personal traits; X21: school image; X22: student expectations; X23: perceived quality; X24: student complaints; X25: student loyalty; X31: other support; X32: family support; X33: friend support; Y11: self-awareness; Y12: social awareness; Y13: nursing professionalism; Y14: the role of nursing work; Y21: self-efficacy; Y22: resilience; Y23: hope; Y24: optimism; Y31: staying in the nursing profession; Y32: leaving the nursing profession.

**Table 3:** Average scores of each variable item, Pearson correlation coefficient, and discriminant validity (*n* = 1332; SD, standard deviation).

	Mean [SD]	Range	X1	X2	X3	Y1	Y2	Y3
Human capital (X1)	3.34 [0.63]	1.06–5.00	**0.809**					
Learning satisfaction (X2)	3.64 [0.63]	1.18–5.00	0.587⁣^∗∗∗^	**0.872**				
Social support (X3)	5.42 [1.08]	1.00–7.00	0.527⁣^∗∗∗^	0.651⁣^∗∗∗^	**0.890**			
Professional values (Y1)	3.99 [0.65]	1.00–5.00	0.478⁣^∗∗∗^	0.617⁣^∗∗∗^	0.635⁣^∗∗∗^	**0.915**		
Psychological capital (Y2)	4.75 [0.77]	1.00–7.00	0.720⁣^∗∗∗^	0.613⁣^∗∗∗^	0.645⁣^∗∗∗^	0.623⁣^∗∗∗^	**0.811**	
Employment intention (Y3)	4.58 [1.30]	1.00–8.00	0.297⁣^∗∗∗^	0.358⁣^∗∗∗^	0.300⁣^∗∗∗^	0.342⁣^∗∗∗^	0.378⁣^∗∗∗^	**0.941**

*Note:* Diagonal bold numbers are AVE square root values, and numbers below the diagonal indicate Pearson correlation coefficients (⁣^∗∗∗^*p* < 0.001). If the AVE square root value is greater than the correlation coefficients of the corresponding variable, it indicates good discriminant validity of the measured variable.

**Table 4:** The fit indexes of the hypothesized model and the modified model.

	Item	Reference standards	Hypothesized model	Modified model
Absolute indexes	GFI	> 0.900	0.858	0.912
	RMSEA	< 0.080	0.087	0.068

Relative indexes	NFI	> 0.900	0.906	0.942
	CFI	> 0.900	0.913	0.949
	TLI	> 0.900	0.898	0.937

**Table 5:** Parameter estimates of variables for the modified model.

Paths	*B* (S.E.)	*β*	*p* value	SMC
Human capital: employment intention	−0.047 (0.143)	−0.009	0.740	0.202
Learning satisfaction: employment intention	0.417 (0.086)	0.129	< 0.001	
Social support: employment intention	−0.101 (0.050)	−0.052	0.045	
Professional values: employment intention	0.630 (0.091)	0.186	< 0.001	
Psychological capital: employment intention	0.594 (0.086)	0.235	< 0.001	
Human capital: psychological capital	0.993 (0.084)	0.499	< 0.001	0.646
Learning satisfaction: psychological capital	0.164 (0.040)	0.128	< 0.001	
Social support: psychological capital	0.234 (0.022)	0.305	< 0.001	
Learning satisfaction: professional values	0.309 (0.030)	0.323	< 0.001	0.566
Social support: professional values	0.162 (0.018)	0.282	< 0.001	
Psychological capital: professional values	0.193 (0.024)	0.258	< 0.001	

*Note: β*: standardized estimate.

Abbreviations: S.E. = standard error, SMC = squared multiple correlation.

**Table 6:** Standardized direct, indirect, and total effects.

Dependent variable	Independent variable	Direct effect	Indirect effect	Total effect
Employment intention	Human capital	−0.009	0.141⁣^∗∗∗^	0.132⁣^∗∗∗^
	Learning satisfaction	0.129⁣^∗∗∗^	0.096⁣^∗∗∗^	0.225⁣^∗∗∗^
	Social support	−0.052⁣^∗^	0.139⁣^∗∗∗^	0.087⁣^∗∗^
	Professional values	0.186⁣^∗∗∗^	—	0.186⁣^∗∗∗^
	Psychological capital	0.235⁣^∗∗∗^	0.048⁣^∗∗∗^	0.283⁣^∗∗∗^

Psychological capital	Human capital	0.499⁣^∗∗∗^	—	0.499⁣^∗∗∗^
	Social support	0.305⁣^∗∗∗^	—	0.305⁣^∗∗∗^
	Learning satisfaction	0.128⁣^∗∗∗^	—	0.128⁣^∗∗∗^

Professional values	Learning satisfaction	0.323⁣^∗∗∗^	0.033⁣^∗∗^	0.356⁣^∗∗∗^
	Social support	0.282⁣^∗∗∗^	0.079⁣^∗∗∗^	0.360⁣^∗∗∗^
	Psychological capital	0.258⁣^∗∗∗^	—	0.258⁣^∗∗∗^
	Human capital	—	0.129⁣^∗∗∗^	0.129⁣^∗∗∗^

⁣^∗^*p* < 0.05.

⁣^∗∗^*p* < 0.01.

⁣^∗∗∗^*p* < 0.001.

## Data Availability

The data used to support the findings of this study have not been made available due to personal privacy and ethical concerns.
